# A new species of *Pristimantis* (Amphibia, Anura, Craugastoridae) from the foothills of the Andes in Manu National Park, southeastern Peru

**DOI:** 10.3897/zookeys.594.8295

**Published:** 2016-05-30

**Authors:** Alexander Shepack, Rudolf von May, Alex Ttito, Alessandro Catenazzi

**Affiliations:** 1 Department of Zoology, Southern Illinois University, Carbondale, IL 62901, USA; 2 Department of Ecology and Evolutionary Biology, University of Michigan, Ann Arbor, USA; 3 Museo de Historia Natural de la Universidad Nacional de San Antonio Abad del Cusco, Cusco, Perú; 4 CORBIDI – Centro de Ornitología y Biodiversidad, Lima, Perú

**Keywords:** Frog, Cusco, Paucartambo, Pristimantis pluvialis, new species

## Abstract

We describe a new species of *Pristimantis* from the humid sub-montane forest of the *Región* Cusco in Peru. *Pristimantis pluvialis* **sp. n.** was collected in the Kosñipata and Entoro valleys at elevations from 740 to 1110 m a.s.l., near the borders of Manu National Park and within the Huachiperi Haramba Queros Conservation Concession. The new species can be distinguished from other members of the genus *Pristimantis* by its rostral tubercle, smooth dorsal skin, and by its advertisement call. *Pristimantis lacrimosus* and *Pristimantis waoranii* superficially most resemble the new species, but *Pristimantis pluvialis* **sp. n.** differs from both species by having a rostral tubercle (absent in *Pristimantis waoranii* and variable in *Pristimantis lacrimosus*) and larger size, from *Pristimantis lacrimosus* by its call emitted at a lower frequency, and from *Pristimantis waoranii* for its dorsal coloration with dark markings. Two other species have partially overlapping distributions and resemble the new species, *Pristimantis mendax* and *Pristimantis olivaceus*, but they produce advertisement calls with much higher dominant frequencies than the advertisement call of the new species. Furthermore, *Pristimantis mendax* differs from the new species by lacking a rostral tubercle and by having a sigmoid inner tarsal fold, whereas *Pristimantis olivaceus* differs by being smaller and by having dorsal skin shagreen with scattered tubercles. The new species has snout-vent length of 21.8–26.9 mm in males (n = 12) and 28.8 mm in a single female.

## Abstract (es)

Describimos una nueva especie de *Pristimantis* de los bosques sub-montanos de la Región Cusco, en Perú. *Pristimantis pluvialis* **sp. n.** fue colectada en los valles de Kosñipata y Entoro a altitudes entre 740 y 1110 m.s.n.m., cerca del límite del Parque Nacional del Manu y dentro de la Concesión para Conservación Huachiperi Haramba Queros. La nueva especie se diferencia de otros miembros del género *Pristimantis* por su tubérculo rostral, piel dorsal lisa, y por su canto de anuncio. Superficialmente, *Pristimantis lacrimosus* y *Pristimantis waoranii* se asemejan a la nueva especie, pero *Pristimantis pluvialis* **sp. n.** difiere de ambas por tener un tubérculo dorsal (ausente en *Pristimantis waoranii* y variable en *Pristimantis lacrimosus*) y mayor tamaño; además, se diferencia de *Pristimantis lacrimosus* por tener un canto de anuncio producido a frecuencias más bajas, y de *Pristimantis waoranii* por su coloración dorsal con manchas oscuras. Dos especies con distribución parcialmente simpátrica se asemejan morfológicamente a la nueva especie, *Pristimantis mendax* y *Pristimantis olivaceus*. Ambas especies producen cantos de anuncio a frecuencias mucho más altas que la nueva especie. Además, *Pristimantis mendax* se distingue de *Pristimantis pluvialis* **sp. n.** por carecer de tubérculo rostral y por tener un pliegue tarsal interno sigmoideo, mientras que *Pristimantis olivaceus* se distingue por su menor tamaño, y por tener piel finamente granular con algunos tubérculos diseminados en la parte dorsal. La nueva especie tiene una longitud hocico-cloaca de 21.8–26.9 mm en machos (n = 12) y de 28.8 mm en una hembra.

## Introduction

The wet tropics are a region of incredibly high biodiversity. The combination of historical, climatic and geographic characteristics foster high speciation rates. In particular, Manu National Park and its surrounding areas have one of the highest herpetofaunal diversity in the world (Catenazzi et al. 2013). Over 155 amphibian species are known from this region, comprising over 2% of known amphibians (Catenazzi et al. 2013). Despite intensive survey efforts, new amphibian species are frequently discovered (Catenazzi et al. 2012; Chaparro et al. 2015; De la Riva et al. 2012).

Manu NP is particularly rich in members of the genus *Pristimantis* (Craugastoridae), as are other regions in the upper Amazon Basin and the eastern slopes of the Andes. This is one of the largest genera of all vertebrates, and is incredibly understudied. It contains nearly 500 species distributed throughout the New World (AmphibiaWeb 2016; Hedges et al. 2008). *Pristimantis* and most members of the Craugastoridae are primarily terrestrial and are generally assumed to be direct-developing, lacking an aquatic tadpole stage (Duellman and Lehr 2009).

A relatively recent divergence and morphological similarities among species may indicate remarkable cryptic diversity within *Pristimantis* (Ortega-Andrade et al. 2015). It can be difficult to discern new species without genetic information, particularly in Peru where this genus is diverse (Aguilar et al. 2010) and counts up to 125 species (AmphibiaWeb 2016). However, surveys sometimes reveal species with unique morphological traits, such is the case of a new, relatively large *Pristimantis* species bearing a rostral tubercle, related to *Pristimantis lacrimosus*, that we discovered during surveys in the Kosñipata Valley near Manu NP and within the Huachiperi Haramba Queros Conservation Concession. Here we describe this new species.

## Methods

The format of the diagnosis, measurements and description follows Duellman and Lehr (2009). Taxonomy follows Hedges et al. (2008), except that we followed Pyron and Wiens (2011) for family placement. Specimens were fixed in 10% formalin and preserved in 70% ethanol. Sex and maturity of specimens were determined by observing sexual characters and gonads through dissections. We measured the following variables to the nearest 0.1 mm with digital calipers under a stereomicroscope: snout–vent length (SVL), tibia length (TL), foot length (FL, distance from proximal margin of inner metatarsal tubercle to tip of Toe IV), head length (HL, from angle of jaw to tip of snout), head width (HW, at level of angle of jaw), eye diameter (ED), tympanum diameter (TY), interorbital distance (IOD), upper eyelid width (EW), internarial distance (IND), and eye–nostril distance (E–N, straight line distance between anterior corner of orbit and posterior margin of external nares). Fingers and toes are numbered preaxially to postaxially from I–IV and I–V respectively. We compared the lengths of toes III and V by adpressing both toes against Toe IV; lengths of fingers I and II were determined by adpressing the fingers against each other. Photographs taken by A. Shepack and A. Catenazzi in the field were used for descriptions of coloration in life, and have been deposited at the Calphotos online database (http://calphotos.berkeley.edu).

We used two sets of recordings to describe the advertisement call. Holotype, CORBIDI 16510 (SVL 24.6 mm; recording #9846 deposited at the Fonoteca Zoológica, Museo Nacional de Ciencias Naturales, Madrid, www.fonozoo.com) was recorded at the type locality at 20:30 on 16 January 2015 (T_air_ = 21.4 °C), along with several unvouchered males. We used a digital recorder (Zoom H2; WAV format, 44 KHz, 24 bit) to record these advertisement calls in 2015. In a second set of recordings, we recorded paratype MUSM 35217 at 21:00 on 2 September 1999 (SVL =22.5 mm; T_air_ = 20.2 °C; recording #9847 deposited at the Fonoteca Zoológica, Museo Nacional de Ciencias Naturales, Madrid), along with several uncaptured males, using a portable cassette recorder (Aiwa HS-F150), a small microphone, and audiotapes.

We used Raven, version 1.4 (Cornell Laboratory of Ornithology) to analyze call length, peak frequency, and calling rate. The Hamming window function for the spectrogram was set at 256 bands. We report means ± SD. We analyzed a total of 380 calls.

We determined the phylogenetic position of the new species with respect to other *Pristimantis* species through analysis of DNA sequence data. Our analysis included the 16S rRNA mitochondrial fragment and the protein-coding gene cytochrome c oxidase subunit I (COI). We used tissue samples from specimens collected in southern Peru (Cusco and Madre de Dios regions) to obtain DNA sequences for the new species and another undescribed *Pristimantis* from the foothills of Manu National Park (Appendix 1). Additionally, we downloaded sequences from Genbank (Appendix 1) of morphologically similar species (*Pristimantis bromeliaceus*, *Pristimantis galdi*, *Pristimantis mendax*, *Pristimantis mindo*, *Pristimantis moro*, *Pristimantis omeviridis*, *Pristimantis schultei*, *Pristimantis subsigillatus*) in the putative *Pristimantis lacrimosus* group (sensu (Arteaga et al. 2013); but see (Padial et al. 2014). Sequences for several other species of *Pristimantis* that possess a rostral tubercle were not available in Genbank. We included the distantly related *Pristimantis ridens* as outgroup (Padial et al. 2014).

Extraction, amplification, and sequencing of DNA followed protocols previously used for *Pristimantis* species (Hedges et al. 2008). We used the 16SA (forward) primer (5’-3’ sequence: CGCCTGTTTATCAAAAACAT) and the 16SB (reverse) primer (5’-3’ sequence: CCGGTCTGAACTCAGATCACGT) to amplify 16S (Palumbi et al. 2002), and we used the dgLCO1490 (forward) primer (GGTCAACAAATCATAAAGAYATYGG) and the dgHCO2198 (reverse) primer (TAAACTTCAGGGTGACCAAARAAYCA) to amplify COI (Meyer et al. 2005). We employed the following thermocycling conditions to amplify DNA using the polymerase chain reaction (PCR): 1 cycle of 96 °C/3 min; 35 cycles of 95 °C/30 s, 55 °C/45 s, 72 °C/1.5 min; 1 cycle 72 °C/7 min. We completed the cycle reactions by using the corresponding PCR primers and the BigDye Terminator 3.1 (Applied Biosystems), and obtained sequence data by running the purified reaction products in an ABI 3730 Sequence Analyzer (Applied Biosystems). We deposited the newly obtained sequences in GenBank (Appendix 1).

We used Geneious R6, v. 6.1.8 (Biomatters, http://www.geneious.com/) to align the sequences with MAFFT, v. 7.017 (Katoh and Standley 2013) alignment program. Prior conducting phylogenetic analysis, we used PartitionFinder, v. 1.1.1 (Lanfear et al. 2012) to select the appropriate models of nucleotide evolution and determined the best partitioning scheme and substitution model for each gene with a Bayesian information criterion (BIC) to. We employed a Maximum Likelihood (ML) approach using RaxML, v. 8.2.4 (Stamatakis 2006) to infer a molecular phylogeny. We used the “f- a” function to conduct a bootstrap analysis and search for the optimal likelihood tree. Our analysis included 20 terminals and a 1064 bp alignment for the concatenated dataset. We used the GTR + Γ model of nucleotide substitution, performed 200 tree searches, and assessed node support using 1000 bootstrap replicates. Additionally, we used the R package APE (Paradis et al. 2004) to estimate uncorrected p-distances (i.e., the proportion of nucleotide sites at which any two sequences are different).

We quantified infection by *Batrachochytrium dendrobatidis*
(Bd) by swabbing frogs with a synthetic dry swab (Medical Wire & Equipment, #113) using a standardized swabbing protocol. Swabs were stroked across the skin a total of 30 times: 5 strokes on each side of the abdominal midline, 5 strokes on the inner thighs of each hind leg, and 5 strokes on the foot webbing of each hind leg (total of 30 strokes/frog). We used a real-time Polymerase Chain Reaction (PCR) assay on material collected on swabs to quantify the level of infection (Boyle et al. 2004). After extraction using PrepMan Ultra, we analyzed DNA amplification in a Life Technologies StepOne Plus qPCR instrument following the protocol outlined in Hyatt et al. (2007) and Boyle et al. (2004), except that extracts were analyzed once. We calculated ZE, the genomic equivalent for Bd zoospores by comparing the qPCR results to a set of standards, and considered any sample with ZE > 1 to be infected or Bd-positive.

Specimens examined are listed in Appendix 2; codes of collections are: CORBIDI = Herpetology Collection, Centro de Ornitología y Biodiversidad, Lima, Peru; KU = Natural History Museum, The University of Kansas, Lawrence, Kansas, USA; MUSM = Museo de Historia Natural Universidad Nacional Mayor de San Marcos, Lima, Peru; MHNG = Muséum d’Histoire Naturelle, Genève, Switzerland; MTD = Museum für Naturkunde Dresden, Dresden, Germany; ROM = Royal Ontario Museum; USNM = National Museum of Natural History (USA).

## Results

### Pristimantis pluvialis sp. n.

Taxon classificationAnimaliaAnuraCraugastoridae

http://zoobank.org/2C675BB5-46BD-4481-BBBF-8D332BD0F562

#### Holotype

**Figure 1.**
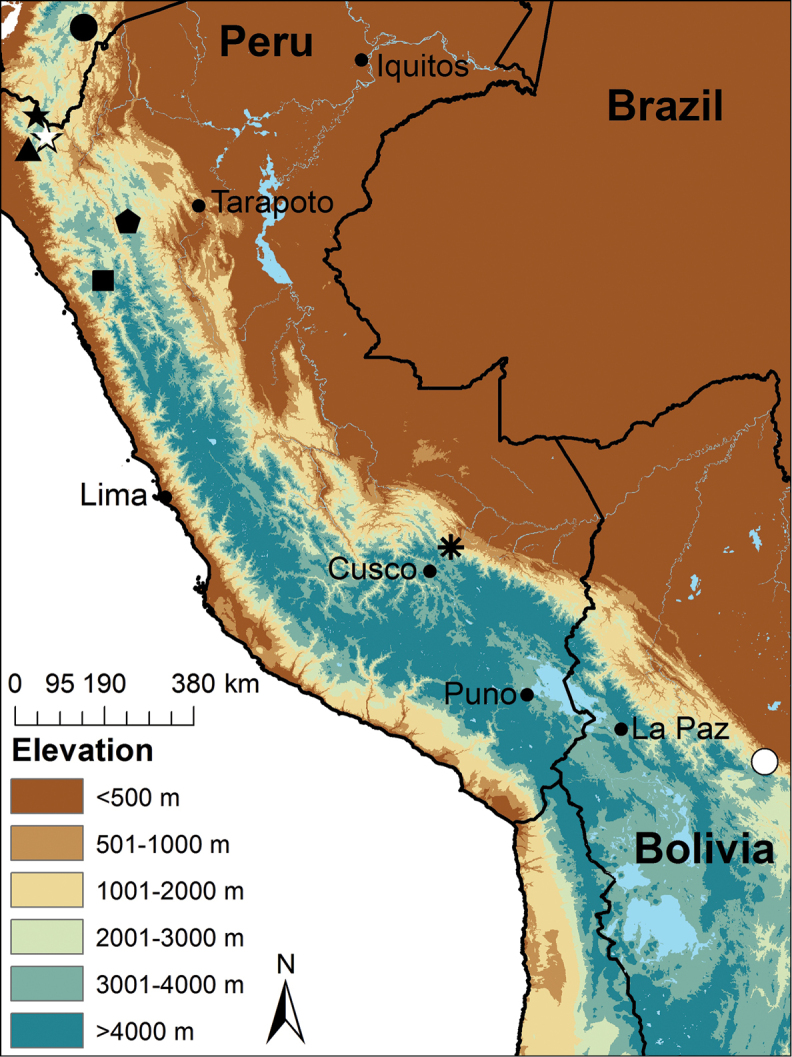
Map of Peru indicating the type localities of Peruvian species of *Pristimantis* known to possess a rostral tubercle or papilla: *Pristimantis proserpens* (black circle), *Pristimantis caeruleonotus* and *Pristimantis coronatus* (black star), *Pristimantis aquilonarius* (white star), *Pristimantis anemerus* (triangle), *Pristimantis corrugatus* (pentagon), *Pristimantis cordovae* (square), *Pristimantis pluvialis* sp. n. (asterisk), and *Pristimantis olivaceus* (white circle).

**Figure 2.**
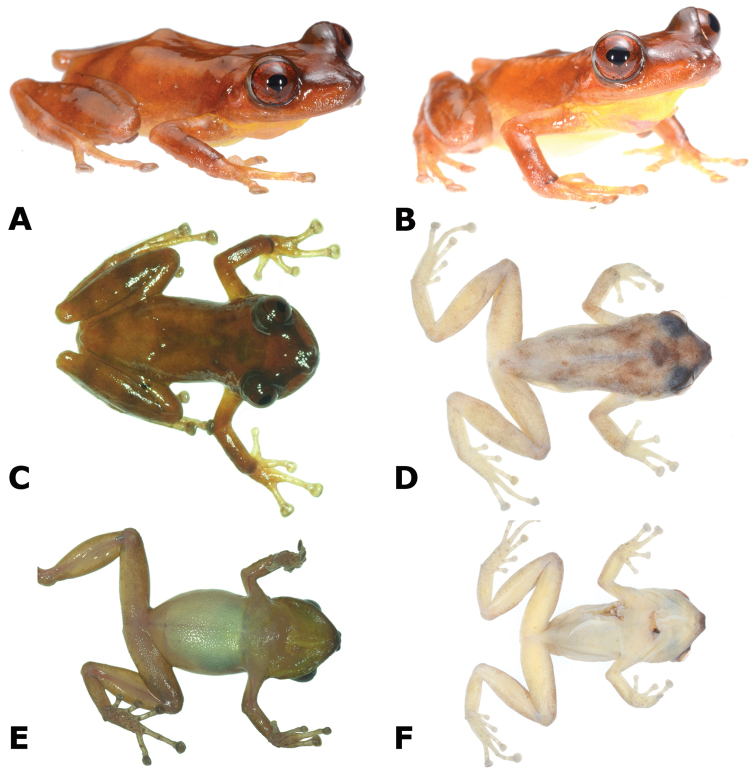
Holotype of *Pristimantis pluvialis* sp. n., male CORBIDI 16510 (SVL 24.6 mm) in dorsolateral view (**A–B**); dorsal (**C–D**) and ventral (**E–F**) views of specimen alive and fixed. Photographs by A. Shepack.

**Figure 3.**
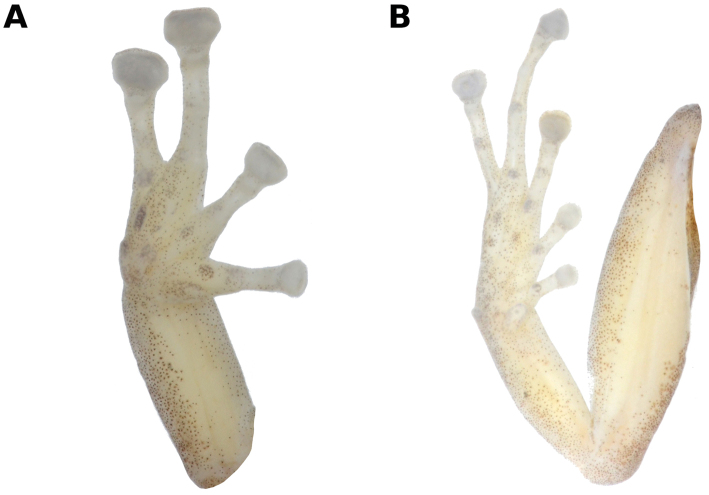
Ventral view of hand and foot of holotype of *Pristimantis pluvialis* sp. n., male CORBIDI 16510 (hand length 5.1 mm, foot length 9.9 mm). Photographs by A. Shepack.

(Figs 1–3). CORBIDI 16510, an adult male from Quitacalzón, 13°01'31.80"S, 71°30'00.72"W (WGS84), , 1050 m a.s.l., Distrito Kosñipata, Provincia Paucartambo, Región Cusco, Peru, collected by A. Shepack, A. Ttito, and A. Catenazzi on 16 January 2015.

#### Paratopotypes

**Figure 4.**
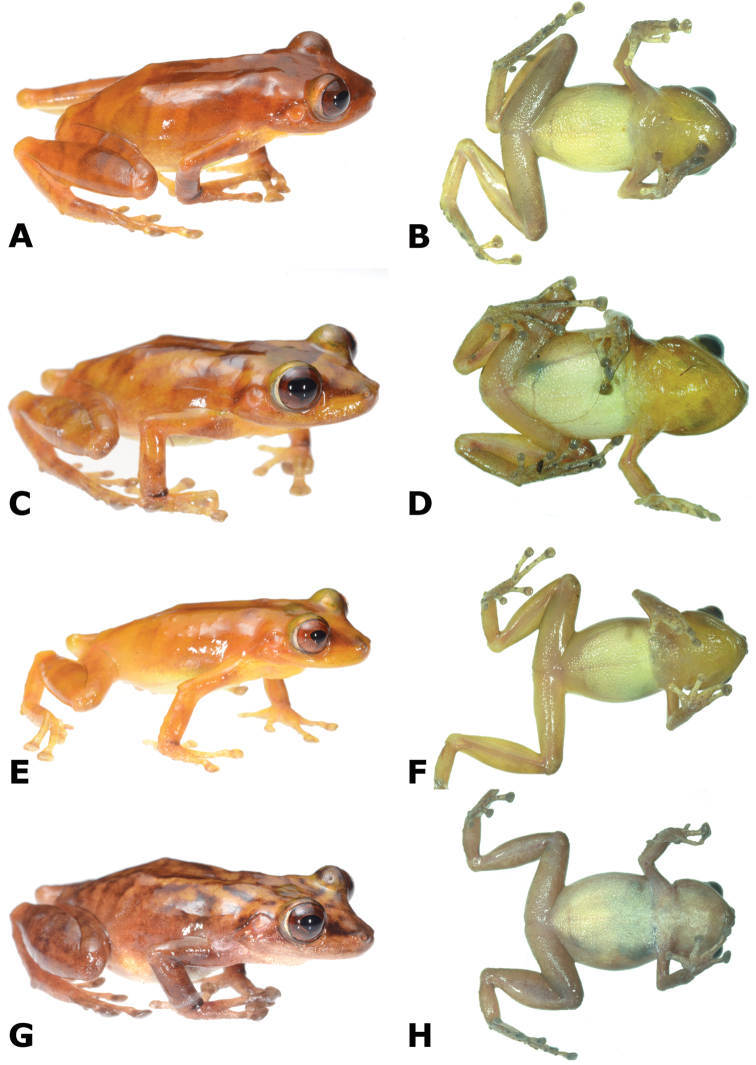
Dorsal and ventral views of *Pristimantis pluvialis* sp. n. paratopotypes; female CORBIDI 16511 (**A–B**); male MHNC 15490 (**C–D**); male CORBIDI 16512 (**E–F**); male MHNC 15489 (**G–H**). Photographs by A. Shepack.

(Fig. 4). CORBIDI 16511, an adult female; CORBIDI 16512 and MHNC 15489–90, two adult males, collected by A. Shepack, A. Ttito, and A. Catenazzi on 16 January 2015.

#### Paratypes

**Figure 5.**
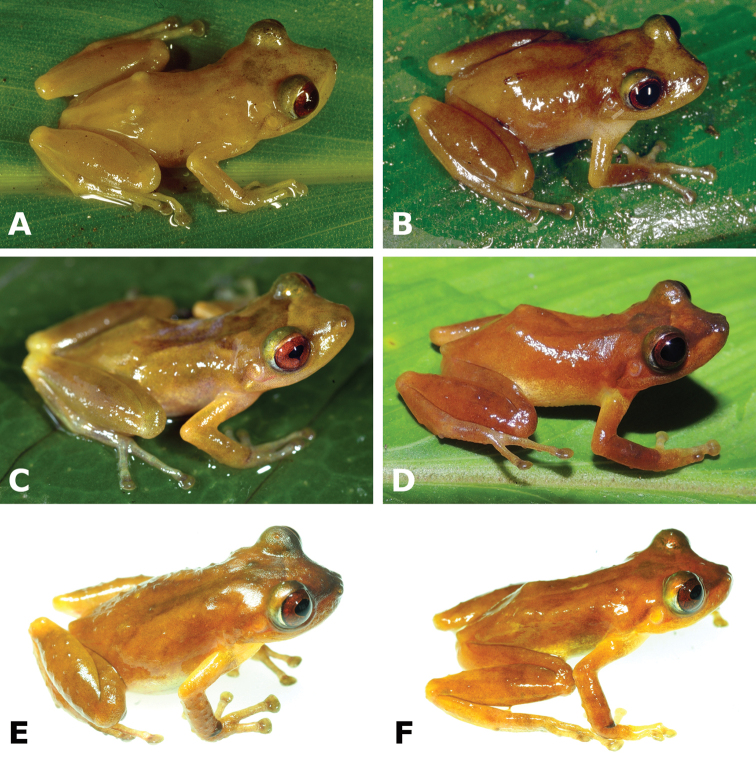
Dorsolateral view of six live male paratypes of *Pristimantis pluvialis* sp. n.: MHNG 2607.11 (**A**), MUSM 35217 (**B**), CORBIDI 11862 (**C**), CORBIDI 16695 (**D**), CORBIDI 17014 (**E**), and CORBIDI 17015 (**F**). Photographs by A. Catenazzi.

(Fig. 5). Eight adult males, all from Distrito Kosñipata: MUSM 35217 and MHNG 2607.12–13 from Río Entoro, 13°00'45"S; 71°21'44"W (WGS84), 740 m a.s.l., collected on 2 September 1999 by A. Catenazzi and R. von May; CORBIDI 11862 from near Chontachaca, 13°01'33"S, 71°29'03"W (WGS84), 930 m a.s.l., collected by A. Catenazzi on 11 August 2012; CORBIDI 17014–15 from near Chontachaca, 13°01'33"S, 71°29'05"W (WGS84), 940 m a.s.l., collected by A. Catenazzi and A. Ttito on 3 March 2016; CORBIDI 16695 from between Chontachaca and Quitacalzón, 13°01'33"S, 71°29'07"W (WGS84), 950 m a.s.l., collected by A. Catenazzi and A. Ttito on 25 January 2014; MHNG 2607.11 from near Radiochayoc, 13°02'07"S, 71°30'46"W (WGS84), 1110 m a.s.l., collected on 25 February 1999 by A. Catenazzi, J. L. Martínez Ruiz and W. Qertehuari Dariquebe.

#### Generic placement.

**Figure 6.**
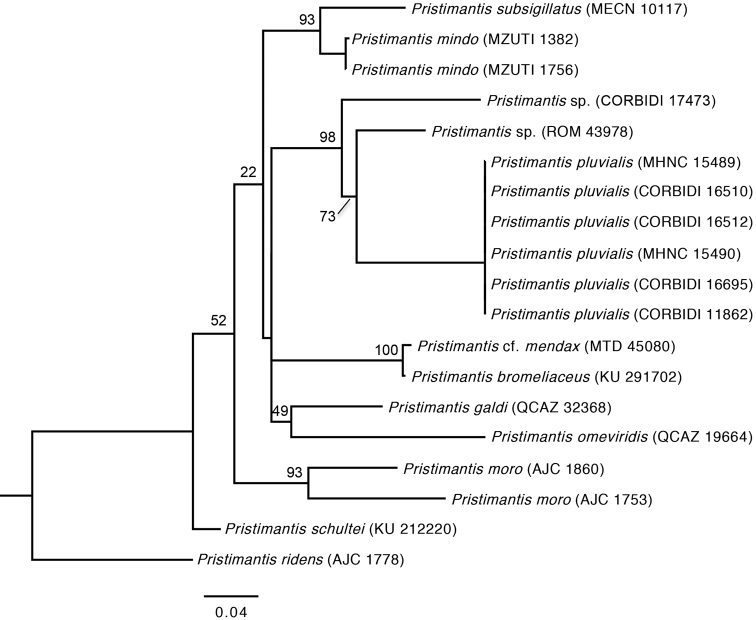
Maximum Likelihood (ML) phylogeny (best tree) based on the combined dataset (16S ribosomal RNA and COI genes). ML bootstrap values are indicated at each node.

We assign this species to *Pristimantis* on the basis of general morphological similarity to other members of the genus and molecular data. The genus *Pristimantis* lacks any diagnostic morphological synapomorphies (Hedges et al. 2008), but molecular phylogenetic analyses support the placement of the new species within the genus (Fig. 6).

#### Diagnosis.

A new species of *Pristimantis* characterized by (1) skin on dorsum smooth, skin on belly areolate, discoidal and dorsolateral folds absent; (2) tympanic membrane differentiated, tympanic annulus distinct; (3) snout moderate in length, with small rostral tubercle, subacuminate in dorsal view and rounded in profile; (4) upper eyelid with minute conical tubercles, narrower than IOD; cranial crests absent; (5) dentigerous process of vomers barely noticeable; (6) vocal slits present; nuptial pads absent; (7) Finger I shorter than Finger II; discs broadly expanded and elliptical; (8) fingers with narrow lateral fringes; (9) single, minute ulnar tubercle present; (10) heel and tarsus lacking tubercles; (11) inner metatarsal tubercle ovoid, of higher relief and about 2.5 times the size of conical, rounded outer metatarsal tubercle; supernumerary plantar tubercles present; (12) toes with narrow lateral fringes; webbing absent; Toe V longer than Toe III; tips of digits expanded, truncate; (13) dorsum beige to reddish-brown with or without dark brown markings; interorbital bar present; venter cream; (14) SVL 21.8–26.9 mm in 12 males, 28.8 mm in one female (Table 1).

**Table 1.** Measurements (in mm) of holotype and paratopotypes of *Pristimantis pluvialis* sp. n. from Quitacalzón, 1050 m a.s.l., Distrito Kosñipata, Provincia Paucartambo, Region Cusco, Peru.

Characters	Holotype, male	Paratopotype, male	Paratopotype, male	Paratopotype, male	Paratopotype, female
	CORBIDI 16510	MHNC 15489	MHNC 15490	CORBIDI 16512	CORBIDI 16511
SVL	24.6	22.8	23.4	23.9	28.8
Tibia length	12.9	13.1	12.9	12.8	15.0
Foot length	9.9	10.9	9.8	9.7	12.9
Head length	8.9	8.8	8.5	8.4	11.3
Head width	8.3	7.6	7.9	7.9	9.8
Interorbital distance	3.3	3.4	3.3	4.0	4.5
Upper eyelid width	2.2	2.2	1.9	1.9	2.2
Internarial distance	1.7	1.8	1.6	1.8	2.1
Eye to nostril distance	2.9	3.0	2.8	2.9	3.5
Snout to eye distance	3.8	3.9	3.7	3.5	4.8
Eye diameter	0.8	1.0	0.8	0.8	1.1
Tympanum diameter	3.0	3.2	3.3	3.3	3.7
Eye to tympanum distance	1.3	1.5	1.5	1.3	1.5
Forearm length	1.0	0.9	0.9	0.8	1.2
Hand length	5.1	5.3	4.9	5.0	6.4
Finger I length	6.6	7.0	6.7	6.4	7.6
Finger II length	2.5	3.0	2.3	2.3	3.1

#### Comparisons.

We tentatively assign *Pristimantis pluvialis* to the putative *Pristimantis lacrimosus* group sensu Arteaga et al. (2013) because of its smooth dorsal skin, presence of rostral tubercle, subacuminate snout profile, moderately long limbs, Finger I shorter than Finger II, expanded digital disks, and distinct tympanic membrane. Furthermore, our phylogenetic analysis (Fig. 6, Tables 2–3) supports the distinctiveness of *Pristimantis pluvialis* from other closely related taxa, including *Pristimantis bromeliaceus*, *Pristimantis galdi*, Pristimantis cf. mendax, *Pristimantis omeviridis*, and two undescribed species (Fig. 6). We found substantial genetic distances (uncorrected p-distances of 0.06–0.15 for 16S and 0.23–0.27 for COI; Tables 2–3) between *Pristimantis pluvialis* and the most closely related species for which mitochondrial sequence data were available. Given our taxon sampling, we emphasize distances for 16S. *Pristimantis pluvialis* is most closely related to two undescribed *Pristimantis*, one from near the type locality (CORBIDI 17473, 16S uncorrected p-distance: 0.06), and another from Guyana (ROM 43978, 16S uncorrected p-distance: 0.07). This species was previously identified as *Pristimantis zeuctotylus* by Hedges et al. (2008), but was treated as *Pristimantis* sp. by Padial et al. (2014). Other closely related species are *Pristimantis moro* (16S uncorrected p-distance: 0.08–0.11), *Pristimantis schultei* (0.10), *Pristimantis bromeliaceus* (0.11), and *Pristimantis mendax* (0.11).

**Table 2.** Uncorrected p-distances of the mitochondrial 16S rRNA gene. Comparisons between *Pristimantis pluvialis* and other taxa are indicated in bold.

	*Pristimantis bromeliaceus* KU291702	*Pristimantis mendax* MTD45080	*Pristimantis schultei* KU212220	*Pristimantis pluvialis* CORBIDI 11862	*Pristimantis pluvialis* CORBIDI 16695	*Pristimantis* sp. CORBIDI 17473	*Pristimantis* sp. ROM 43978	*Pristimantis mindo* MZUTI 1382	*Pristimantis mindo* MZUTI 1756	*Pristimantis subsigillatus* MECN 10117	*Pristimantis galdi* QCAZ32368	*Pristimantis moro* AJC1753	*Pristimantis moro* AJC1860	*Pristimantis omeviridis* QCAZ19664	*Pristimantis ridens* AJC1778
*Pristimantis bromeliaceus* KU291702	0.00														
*Pristimantis mendax* MTD45080	0.01	0.00													
*Pristimantis schultei* KU212220	0.08	0.08	0.00												
*Pristimantis pluvialis* CORBIDI 11862	**0.11**	**0.11**	**0.10**	**0.00**											
*Pristimantis pluvialis* CORBIDI 16695	**0.11**	**0.11**	**0.10**	**0.00**	**0.00**										
*Pristimantis* sp. CORBIDI 17473	0.10	0.10	0.07	**0.06**	**0.06**	0.00									
*Pristimantis* sp. ROM 43978	0.12	0.11	0.09	**0.07**	**0.07**	0.07	0.00								
*Pristimantis mindo* MZUTI 1382	0.09	0.09	0.08	**0.10**	**0.10**	0.09	0.09	0.00							
*Pristimantis mindo* MZUTI 1756	0.09	0.09	0.08	**0.10**	**0.10**	0.08	0.09	0.00	0.00						
*Pristimantis subsigillatus* MECN 10117	0.12	0.11	0.09	**0.11**	**0.11**	0.10	0.10	0.06	0.05	0.00					
*Pristimantis galdi* QCAZ32368	0.10	0.10	0.09	**0.11**	**0.12**	0.10	0.10	0.08	0.07	0.11	0.00				
*Pristimantis moro* AJC1753	0.09	0.10	0.07	**0.11**	**0.11**	0.10	0.10	0.08	0.08	0.09	0.09	0.00			
*Pristimantis moro* AJC1860	0.08	0.08	0.06	**0.08**	**0.08**	0.08	0.09	0.07	0.07	0.08	0.09	0.05	0.00		
*Pristimantis omeviridis* QCAZ19664	0.12	0.12	0.11	**0.13**	**0.13**	0.12	0.14	0.12	0.12	0.12	0.11	0.10	0.10	0.00	
*Pristimantis ridens* AJC1778	0.14	0.15	0.12	**0.14**	**0.15**	0.15	0.17	0.14	0.14	0.16	0.16	0.14	0.12	0.14	0.00

**Table 3.** Uncorrected p-distances of the protein-coding gene cytochrome c oxidase subunit I (COI). Comparisons between *Pristimantis pluvialis* and other taxa are indicated in bold.

	*Pristimantis moro* AJC1753	*Pristimantis moro* AJC1860	*Pristimantis pluvialis* CORBIDI 16695	*Pristimantis pluvialis* CORBIDI 16510	*Pristimantis pluvialis* MHNC 15489	*Pristimantis pluvialis* MHNC 15490	*Pristimantis pluvialis* CORBIDI 11862	*Pristimantis pluvialis* CORBIDI 16512	*Pristimantis* sp. CORBIDI 17473	*Pristimantis ridens* AJC1778
*Pristimantis moro* AJC1753	0.00									
*Pristimantis moro* AJC1860	0.18	0.00								
*Pristimantis pluvialis* CORBIDI 16695	**0.23**	**0.23**	**0.00**							
*Pristimantis pluvialis* CORBIDI 16510	**0.23**	**0.23**	**0.00**	**0.00**						
*Pristimantis pluvialis* MHNC 15489	**0.23**	**0.23**	**0.00**	**0.00**	**0.00**					
*Pristimantis pluvialis* MHNC 15490	**0.23**	**0.23**	**0.00**	**0.00**	**0.00**	**0.00**				
*Pristimantis pluvialis* CORBIDI 11862	**0.23**	**0.23**	**0.00**	**0.00**	**0.00**	**0.00**	**0.00**			
*Pristimantis pluvialis* CORBIDI 16512	**0.23**	**0.23**	**0.00**	**0.00**	**0.00**	**0.00**	**0.00**	**0.00**		
*Pristimantis* sp. CORBIDI 17473	0.26	0.24	**0.23**	**0.23**	**0.23**	**0.23**	**0.23**	**0.23**	0.00	
*Pristimantis ridens* AJC1778	0.26	0.24	**0.26**	**0.26**	**0.27**	**0.26**	**0.26**	**0.26**	0.30	0.00

The new species differs from most known Peruvian species of *Pristimantis* by having a rostral tubercle. Fewer than 20 species of Peruvian *Pristimantis* possess a rostral papilla or tubercle (Duellman and Lehr 2009): *Pristimantis acuminatus*, *Pristimantis aquilonaris*, *Pristimantis bromeliaceus*, *Pristimantis caeruleonotus*, *Pristimantis cordovae*, *Pristimantis coronatus*, *Pristimantis lacrimosus* (variable), *Pristimantis olivaceus*, *Pristimantis omeviridis*, *Pristimantis pardalinus*, and *Pristimantis proserpens*, *Pristimantis rhodostichus*, and *Pristimantis schultei*. *Pristimantis pluvialis* differs from all these species by having smooth dorsal skin and by its larger snout-vent length reaching 24.9 mm in males (except for *Pristimantis cordovae*, the largest *Pristimantis* bearing a rostral tubercle, and whose males reach 27.1 mm in SVL).

The two species that superficially most resemble *Pristimantis pluvialis* are *Pristimantis lacrimosus* and *Pristimantis waoranii*. However, *Pristimantis pluvialis* differs from both species by having a rostral tubercle (absent in *Pristimantis waoranii* and variable in *Pristimantis lacrimosus*), and by its larger size. Furthermore, it differs from *Pristimantis lacrimosus* by its call with lower dominant frequency (~2500 Hz). Calls of *Pristimantis lacrimosus* available at AmphibiaWeb Ecuador (Read 2012; Ron et al. 2016) have higher dominant frequency ranging from 3100–3273 Hz (n = 6). Furthermore, the new species differs from *Pristimantis waoranii* by having dark bands or markings on the dorsum (absent in *Pristimantis waoranii*). Another morphologically similar species, *Pristimantis schultei*, has an acuminate snout in dorsal view (subacuminate in *Pristimantis pluvialis*), skin on dorsum shagreen (generally smooth), and heel and outer edge of tarsus bearing many low tubercles (tubercles absent). Furthermore, *Pristimantis schultei* occurs in northern Peru at elevations above 2400 m (below 1110 m for *Pristimantis pluvialis*), and its advertisement call consists of a double note, “ping-ping” (Duellman 1990), in contrast with the single, low frequency note produced by *Pristimantis pluvialis*.

Two species related to *Pristimantis lacrimosus*, *Pristimantis mendax* and *Pristimantis olivaceus*, occur near the type locality of *Pristimantis pluvialis* in Manu NP and surrounding areas in southern Peru (Catenazzi et al. 2013; Duellman 1978b; Duellman and Lehr 2009; Köhler et al. 1998). In addition to the characters listed in the previous paragraph, *Pristimantis olivaceus* further differs from *Pristimantis pluvialis* by being smaller (17.7–22.1 mm in males, and up to 25.5 mm in females; Duellman and Lehr 2009) and by having dorsal skin shagreen with scattered tubercles and dorsal coloration brownish green or olive green. Both species produce advertisement calls with higher dominant frequencies (4000–5320 Hz; see FonoZoo recording #875 for *Pristimantis mendax*, and Köhler et al. 1998 and Márquez et al. 2002 for *Pristimantis olivaceus*) than the advertisement call of the new species (~2500 Hz). *Pristimantis mendax* further differs from *Pristimantis pluvialis* by lacking a rostral tubercle, by possessing a sigmoid inner tarsal fold and by having dorsal skin shagreen with scattered spicules. Furthermore, *Pristimantis mendax* occurs in montane cloud forests above 1400 m (Duellman and Lehr 2009), an elevational distribution range that does not appear to overlap with that of *Pristimantis pluvialis*.

#### Description of holotype.

Adult male (24.6 mm SVL); head narrower than body, its length 36.3% of SVL; head slightly longer than wide; head width 33.6% of SVL; snout short, squared in dorsal view, subtruncate in lateral view (Fig. 2); eye large, 33.9% of head length, its diameter 0.97 times its distance from the nostril; nostrils slightly protuberant, situated close to snout; canthus rostralis weakly concave in dorsal view, rounded in profile; loreal area flat; lips rounded; dorsal surface of head smooth and upper eyelids with minute tubercles; upper eyelid width 65.7% of interorbital distance; supratympanic fold absent; tympanic membrane not differentiated, tympanic annulus visible; postrictal ridges or tubercles absent. Choanae round, very small, positioned far anterior and laterally, widely separated from each other, not concealed by palatal shelf of maxilla; dentigerous processes of vomer and vomerine teeth barely noticeable.

Skin on dorsum smooth; no dorsolateral folds; skin on flanks smooth; skin on ventral surfaces and gular regions areolate; pectoral and discoidal folds absent; cloacal sheath absent, cloaca not protuberant; cloacal region lacking tubercles. Ulnar tubercle present, minute; palmar tubercle flat and bifurcate, its inner lobe much larger than outer lobe; palmar tubercle approximately twice the size of elongate, thenar tubercle; supernumerary palmar tubercles present; subarticular tubercles prominent, ovoid in ventral view, rounded in lateral view; fingers with narrow lateral fringes; fingers length when adpressed, 3 > 4 > 2 > 1 (Fig. 3); tips of digits broadly expanded and elliptical, pads with well-defined circumferential grooves (Fig. 3); forearm without tubercles.

Tibia length 52.5% of SVL; foot length 40.3% of SVL; upper and posterior surfaces of hindlimbs smooth; heel without tubercles; outer surface of tarsus without tubercles; inner metatarsal tubercle ovoid, of higher relief and about 2.5 times the size of conical, rounded outer metatarsal tubercle; supernumerary plantar tubercles present; subarticular tubercles rounded, ovoid in dorsal view; toes with narrow lateral fringes, basal webbing absent; discs of toes expanded, rounded; toes with ventral pads well-defined by circumferential grooves; toe lengths, when adpressed, 4 > 5> 3 > 2 > 1 (Fig. 3).

Measurements of holotype and paratopotypes are provided in Table 1. The SVL of paratypes (all males) are (in mm): MUSM 35217 = 22.5, MHNG 2607.11= 24.2, MHNG 2607.12 = 21.8, MHNG 2607.13 = 24.2, CORBIDI 11862 = 22.9, and CORBIDI 16695 = 24.9.

#### Coloration of holotype in life.

Dorsum orange-brown with faint brown markings (Fig. 2). Interorbital bar dark brown, forming a triangular shape posteriorly; canthus rostralis dark brown; light green on upper eyelids. Triangular brown patterning on back, not extending to venter. Hind legs with broad brown barring. Forelimbs with faint brown barring. Throat yellowish-cream; venter cream.

#### Coloration of holotype in alcohol.

Dorsal surfaces of head, body, and limbs grayish-tan, with dark brown regions around scapulae (see Fig. 2). Interorbital as a dark blotch that extends posteriorly; canthus rostralis dark brown. Dorsal surfaces of hind limbs with dark flecking. Iris dark gray. Throat pale white; chest and belly pale white to cream; ventral surfaces of thighs the same color as chest and belly; plantar and palmar surfaces and tips of digits pale, tubercles darker gray.

#### Variation.

Coloration in life is based on field notes and photographs taken by A. Shepack and A. Catenazzi of 13 type specimens. The dorsum is beige to reddish-brown with or without faint dark-brown markings (Figs 3–4). A dark brown interorbital bar is present in most specimens (barely visible in some individuals). The iris is bronze with dark-brown to red reticulations. Some individuals possess faint brown barring on hind legs. The throat is cream to yellowish-white while the belly is predominantly cream to white. Dorsal skin is generally smooth, but CORBIDI 16695, 17014 (Fig. 5), and MHNG 2607.13, have minute, scattered tubercles, indicating that skin texture might be a variable trait. Some individuals have small tubercles on the outer edge of tarsus.

#### Vocalization and reproduction.

**Figure 7.**
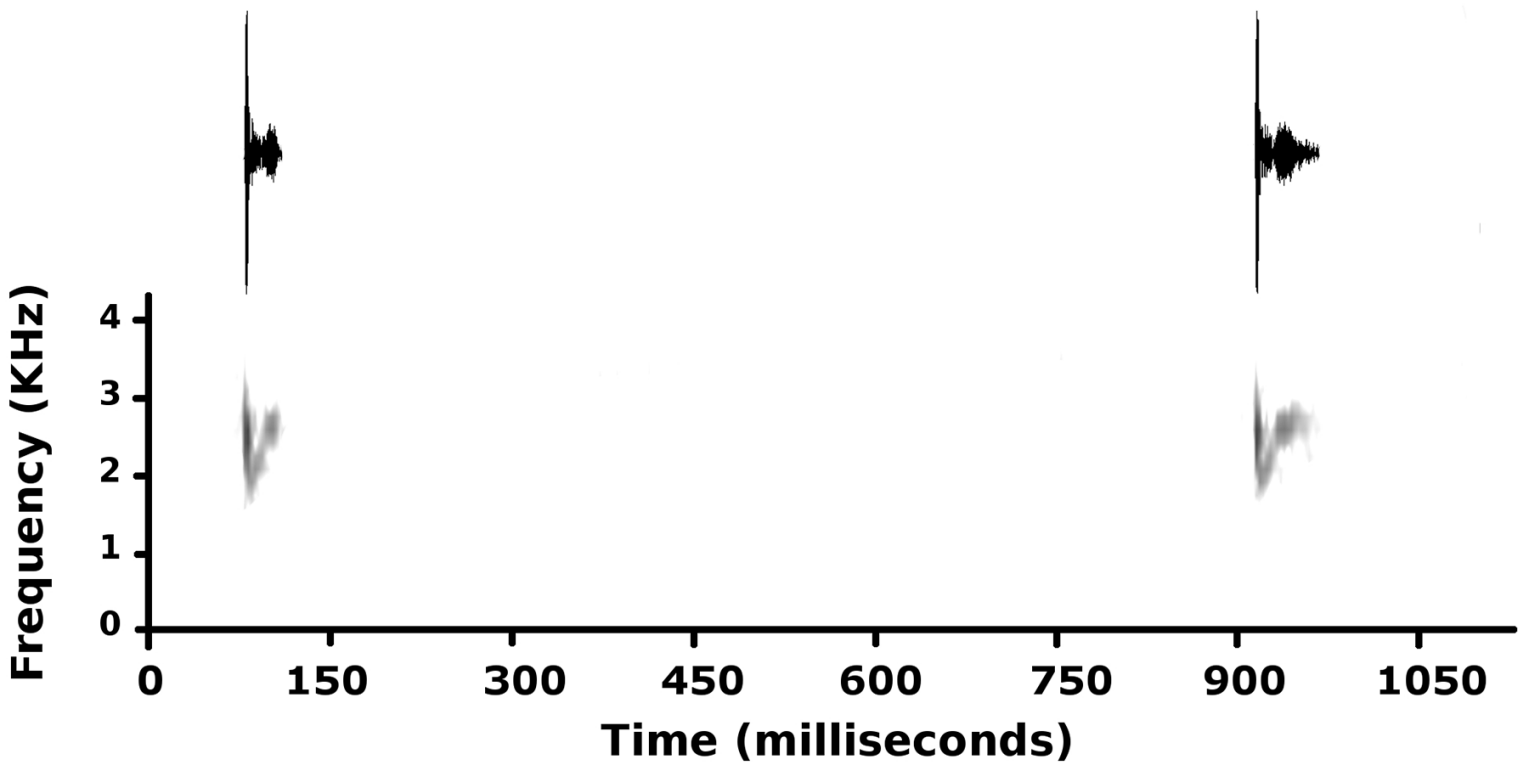
Advertisement call (two calls) of male CORBIDI 16510 (SVL 24.6 mm), holotype of *Pristimantis pluvialis* sp. n., recorded at the type locality on 16 January 2015 (T_air_ = 21.4°C).

Males call from grasses, shrubs, and trees in the understory of the submontane forest, during crepuscular hours and at night, conspicuously after heavy rains. Holotype CORBIDI 16510 was calling from a broad fern leaf at 150 cm above the ground, along a trail at ~30 m from a stream (T_air_ = 21.4°C). The advertisement call consisted of a note 28.7 ± 0.7 ms (range 23.0–35.0 ms, n = 20) in duration (Fig. 7). Pulses emitted at the highest amplitude had dominant frequencies of 2412–2584 Hz (average 2489 ± 20 Hz, n = 20) and were located in the first half of the note (Fig. 7). The calling rate was 0.70 calls/second at a temperature of 21.4°C. Male MUSM 35217 was perched on a shrub at 2 m, near a stream (T_air_ = 20.2°C), and produced single note calls 36.0 ± 0.5 ms (range 24.0–58.0 ms, n = 102) in duration, with dominant frequencies of 2067–2756 Hz (average 2407 ± 19 Hz, n = 102), at a calling rate of 0.64 calls/s. At least three unvouchered males were recorded at the type locality near the holotype (T_air_ = 21.4°C). Their calls were 40.7 ± 0.2 ms (range 26.0–47.0 ms, n = 220) in duration, with dominant frequencies of 2067–2756 Hz (average 2586 ± 20 Hz, n = 220); call rate could not be determined. Similarly, several unvouchered males recorded near MUSM 35217 (T_air_ = 20.2°C) produced calls with dominant frequencies of 2067–2584 Hz (average 2312 ± 13 Hz, n = 104), but their note durations and call rates could not be determined. Overall, the call of the new species can be described as a single “tock” note, 23–58 ms in duration, emitted at a rate of 0.64–0.70 calls/s, and with peak frequency ranging from 2312–2756 Hz. The call has amplitude and frequency modulation (Fig. 7): a short, high energy pulse with frequency decreasing from dominant frequency (~2500 Hz, see above) to ~2000 Hz is followed by a low energy pulse with frequency increasing from ~2000 Hz to ~2500 Hz.

#### Etymology.

The name of the new species is a Latin word and refers to the high rainfall recorded at the type locality, which averages ~6 m annually, and represents the peak rainfall amount along the elevational transition from the Amazon lowlands to the Andean peaks. Furthermore, males of *Pristimantis pluvialis* typically call during or immediately after heavy rains.

#### Distribution, natural history, and threats.

**Figure 8.**
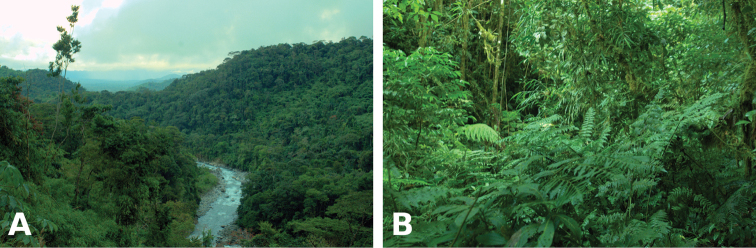
Habitat of *Pristimantis pluvialis* sp. n. at 1050 m a.s.l. Males and females were found on vegetation between 1–2 m above the ground.

The new species was found during surveys in the humid sub-montane forests of the Kosñipata and Entoro valleys (Fig. 8). Observers made intensive visual searches of vegetation and leaf litter during evenings (18h30–0h00). Individuals were found after rains, calling on vegetation up to 2 m above the ground. Male CORBIDI 16511 and female CORBIDI 16512 were captured while in amplexus, during one such choruses on 16 January 2015. Average individual mass was 0.97 g ± 0.06 for males (n = 12) and 2.1 g for one female. The oviducts of this female contained 22 unpigmented eggs, about 2.5 mm in diameter. Additionally, four out of ten individuals (MHNC 15490 and CORBIDI 11862, 16512 and 16695) tested positive for the amphibian chytrid fungus *Batrachochytrium dendrobatidis*. This fungus has been implicated in population declines of numerous other species in this region, although it is unknown what effect it has had on this species, and there is no evidence for declines in the populations of *Pristimantis pluvialis* (Catenazzi et al. 2011; Catenazzi et al. 2014). In addition to being found near, and likely within, Manu NP, *Pristimantis pluvialis* has been found within the Huachiperi Haramba Queros Conservation Concession, a protected area consisting of state-owned lands given in concession to private organizations with the goal of preserving biodiversity. The Huachiperi Haramba Queros concession, legally recognized in 2006 was the first concession to be granted to an indigenous community in Peru. Sympatric frog species at the type locality include *Cochranella nola*, *Hypsiboas gladiator*, *Osteocephalus mimeticus*, *Pristimantis platydactylus*, *Pristimantis reichlei*, and *Rulyrana spiculata*. Other species found around the type locality are *Allobates alessandroi*, *Ameerega simulans*, *Dendropsophus parviceps*, *Hyalinobatrachium bergeri*, *Hyloscirtus phyllognathus*, *Hypsiboas lanciformis*, *Noblella* sp., *Oreobates granulosus*, *Pristimantis danae*, *Pristimantis fenestratus*, *Pristimantis mendax*, *Pristimantis toftae*, *Ranitomeya sirensis*, *Rhinella leptoscelis*, *Rhinella margaritifera*, *Rhinella tacana*, *Rulyrana spiculata* and *Scinax ruber*.

## Discussion

Our phylogenetic analysis indicates that *Pristimantis pluvialis* is most closely related to two undescribed *Pristimantis* species, one from the same valley near the type locality of *Pristimantis pluvialis*, and one from Guyana (Fig. 6; Padial et al. 2014). The species from Guyana (*Pristimantis* sp. ROM 43978) had previously been identified as *Pristimantis zeuctotylus* (Hedges et al. 2008). Furthermore, four other species, *Pristimantis omeviridis*, *Pristimantis galdi*, *Pristimantis mindo*, and *Pristimantis subsigillatus*, are closely related to *Pristimantis pluvialis* (Fig. 6). The most comprehensive molecular phylogenetic study of terraranas to date also found that most of these taxa form one clade (Padial et al. 2014). Nevertheless, given that there was low resolution in some nodes, both in the study by Padial et al. (2014) and this study, analyses including additional genes are needed to further examine the relationships among species in this group.

The morphologically similar *Pristimantis lacrimosus* presumably is a complex of at least two species, formed by lowland populations of small-sized individuals around the type locality in Ecuador, and larger individuals from the Andean slopes (Duellman 1978a; Lynch and Duellman 1980), which possibly form a distinct and still unrecognized species. *Pristimantis pluvialis* is larger, calls at lower frequency, and occurs at higher elevations than *Pristimantis lacrimosus*. Furthermore, the locality of the neotype of *Pristimantis lacrimosus*, collected at Santa Cecilia (Heyer and Hardy 1991), is 1600 km NW of the type locality of *Pristimantis pluvialis*, a very long distance considering the degree of endemism in montane and sub-montane *Pristimantis*. We lack sequences for *Pristimantis waoranii*, but the absence of rostral tubercle and dark dorsal markings in this species differentiates it from all individuals of *Pristimantis pluvialis* in our type series.

*Pristimantis pluvialis* is morphologically similar to *Pristimantis olivaceus*, which has previously been reported from Manu NP (Duellman and Lehr 2009) and southeastern Peru (Köhler et al. 1998). In light of our present description, a closer examination of existing museum specimens, along with DNA sequence data and call recordings for new observations of putative *Pristimantis olivaceus* from southeastern Peru, are needed to confirm assignment of museum specimens to this species, and to properly identify newly collected specimens. Of the characters we have mentioned in the diagnosis and comparisons, skin texture should be used with caution, because previous work shows that this trait is variable in some *Pristimantis* (Guayasamin et al. 2015), and because three of our types, despite not displaying the shagreen skin texture seen in *Pristimantis olivaceus*, differ from other smooth-skin types by having minute, scattered tubercles on dorsal surfaces.

*Pristimantis pluvialis* and *Pristimantis olivaceus* are most easily distinguished by their overall coloration, which is reddish-brown in *Pristimantis pluvialis* and green in *Pristimantis olivaceus*, by the larger size of *Pristimantis pluvialis* (SVL of males <22 mm in *Pristimantis olivaceus*, and >22 mm in *Pristimantis pluvialis*), and most notably by differences in their advertisement calls. Both species produce calls consisting of short, single notes, but whereas *Pristimantis pluvialis* emits “tocks” with a dominant frequency at ~2500 Hz during an initial burst followed by downward frequency modulation in the middle of the call, *Pristimantis olivaceus* emits “chirps” with a dominant frequency at ~4900 Hz with increasing frequency during the call but little amplitude modulation (Köhler et al. 1998).

With the addition of *Pristimantis pluvialis* the genus *Pristimantis* now contains 474 known species (AmphibiaWeb 2016). This cryptically diverse group surely contains even more undescribed species (Ortega-Andrade et al. 2015). We discovered *Pristimantis pluvialis* in a region where multiple researchers, including the authors, have worked previously. This suggests that continued surveying efforts are necessary to achieve a full understanding of herpetological diversity in this area (Catenazzi et al. 2013; Catenazzi and von May 2014).

## Supplementary Material

XML Treatment for Pristimantis pluvialis

## Appendix 1

### Gene sequences for molecular analyses

**Table:** GenBank accession numbers for the taxa and genes sampled in this study. ^A^*Pristimantis* sp. (ROM 43978) was previously identified as *Pristimantis zeuctotylus* by Hedges et al. (2008a), but is treated herein as *Pristimantis* sp. following Padial et al. (2014).

Taxon	Voucher Nbr.	16S	COI
*Pristimantis omeviridis*	QCAZ19664	EU130579	—
*Pristimantis bromeliaceus*	KU 291702	EF493351	—
*Pristimantis galdi*	QCAZ 32368	EU186670	—
Pristimantis cf. mendax	MTD45080	EU186659	—
*Pristimantis mindo*	MZUTI 1382	KF801584	—
*Pristimantis mindo*	MZUTI 1756	KF801581	—
*Pristimantis moro*	AJC 1860	JN991454	JN991384
*Pristimantis moro*	AJC 1753	JN991453	JN991383
*Pristimantis pluvialis* sp.n.	CORBIDI 11862	KX155577	KX155580
*Pristimantis pluvialis* sp.n.	CORBIDI 16510	—	KX155581
*Pristimantis pluvialis* sp.n.	CORBIDI 16512	—	KX155582
*Pristimantis pluvialis* sp.n.	CORBIDI 16695	KX155578	KX155583
*Pristimantis pluvialis* sp.n.	MHNC 15489	—	KX155584
*Pristimantis pluvialis* sp.n.	MHNC 15490	—	KX155585
*Pristimantis* sp.	CORBIDI 17473	KX155579	— KX159303
*Pristimantis ridens*	AJC 1778	KR863320	KR863063
*Pristimantis schultei*	KU 212220	EF493681	—
*Pristimantis subsigillatus*	MECN 10117	KF801580	—
*Pristimantis* sp.^A^	ROM 43978	EU186678	—

## Appendix 2

### Specimens examined

*Pristimantis acuminatus* (5 specimens): PERU: Amazonas: Quebrada Kampankis, CORBIDI 11388, 11403; Cusco: Cashiriari-3, S of Río Camisea, USNM 537763; Pagoreni, Río Camisea, USNM 537764; San Martín-3, ~5 km N Río Camisea, USNM 537762.

*Pristimantis bromeliaceus* (8 specimens): PERU: Amazonas: Chonza Alta, Bagua, CORBIDI 778; Pasco: Comunidad Campesina Chacos, CORBIDI 3859; San Martín: Abra Patricia, CORBIDI 510–12, 516–17; Quintecocha, MUSM 24448–49.

*Pristimantis lacrimosus* (10 specimens): PERU: Loreto: Sierra del Divisor, CORBIDI 3941; Río Tapiche, CORBIDI 12133–38; Campamento Piedras, Putumayo, CORBIDI 5894, 5899, 5903.

*Pristimantis mendax* (6 specimens): PERU: Cusco: Paucartambo: Cusco-Pilcopata road, 1480 m., AMNH 157016; USNM 345921, 346336; MUSM 21105–07.

*Pristimantis olivaceus* (14 specimens): PERU: Cusco: Cashiriari-2, ~4 km S of Río Camisea, USNM 538039–43; Cashiriari-3, S of Río Camisea, USNM 537805; Konkariari Creek Camp, Río Urubamba, USNM 538044–45; Comunidad Nativa Puyentimari, CORBIDI 8296, 9765–66, Kinteroni, CORBIDI 10260; Madre de Dios: Colpa de Guacamayos, Río Tambopata, USNM 332440; Pakitza, PN Manu, USNM 342614–15.

*Pristimantis rhodostichus* (2 specimens): PERU: Amazonas: Cabeceras Katerpiza, CORBIDI 9441; Loreto: Cabeceras Wee, CORBIDI 11430.

*Pristimantis schultei* (21 specimens): PERU: Amazonas: Laguna de los Cóndores, MUSM 23040–48; ACP Huiquilla, CORBIDI 368; Yuramarca, CORBIDI 452–62.
